# Conditional knockout of *PDK1* in osteoclasts suppressed osteoclastogenesis and ameliorated prostate cancer-induced osteolysis in murine model

**DOI:** 10.1186/s40001-023-01425-8

**Published:** 2023-10-13

**Authors:** Yanan Zhang, Haibin Nong, Yiguang Bai, Quan Zhou, Qiong Zhang, Mingfu Liu, Pan Liu, Gaofeng Zeng, Shaohui Zong

**Affiliations:** 1 Guangxi Collaborative Innovation Center for Biomedicine, Guangxi Medical University, Nanning, China; 2 Department of Spine Osteopathia, The First Affiliated Hospital of Guangxi Medical University, Nanning, China; 3 College of Public Hygiene of Guangxi Medical University, Nanning, China; 4 Department of Orthopaedics, Nanchong Central Hospital, The Second Clinical Institute of North Sichuan Medical College, Nanchong, China; 5 Research Centre for Regenerative Medicine and Guangxi Key Laboratory of Regenerative Medicine, Guangxi Medical University, Nanning, Guangxi China

**Keywords:** *PDK1*, Prostate cancer, Invasion, Osteoclastogenesis, Osteolysis

## Abstract

**Background:**

The development and maintenance of normal bone tissue is maintained by balanced communication between osteoblasts and osteoclasts. The invasion of cancer cells disrupts this balance, leading to osteolysis. As the only bone resorbing cells in vivo, osteoclasts play important roles in cancer-induced osteolysis. However, the role of 3-phosphoinositide-dependent protein kinase-1 (*PDK1*) in osteoclast resorption remains unclear.

**Methods:**

In our study, we used a receptor activator of nuclear factor-kappa B (RANK) promoter‐driven Cre‐LoxP system to conditionally delete the *PDK1* gene in osteoclasts in mice. We observed the effect of osteoclast‐specific knockout of *PDK1* on prostate cancer-induced osteolysis. Bone marrow-derived macrophage cells (BMMs) were extracted and induced to differentiate osteoclasts in vitro to explore the role of PDK1 in osteoclasts*.*

**Results:**

In this study, we found that *PDK1* conditional knockout (cKO) mice exhibited smaller body sizes when compared to the wild-type (WT) mice. Moreover, deletion of *PDK1* in osteoclasts ameliorated osteolysis and r*PDK1*educed bone resorption markers in the murine model of prostate cancer-induced osteolysis. In vivo, we discovered that osteoclast‐specific knockout of suppressed RANKL-induced osteoclastogenesis, bone resorption function, and osteoclast-specific gene expression (*Ctsk*, *TRAP*, *MMP-9*, *NFATc1*). Western blot analyses of RANKL-induced signaling pathways showed that conditional knockout of *PDK1* in osteoclasts inhibited the early nuclear factor κB (NF-κB) activation, which consequently suppressed the downstream induction of NFATc1.

**Conclusion:**

These findings demonstrated that *PDK1* performs an important role in osteoclastogenesis and prostate cancer-induced osteolysis by modulating the *PDK1*/AKT/NF-κB signaling pathway.

## Introduction

Bone tissue is the most common site of metastatic prostate cancer and breast cancer, and 70% of patients with metastatic prostate cancer have bone metastasis [1]. Once cancer cells invade the bone, patients are rarely cured and metastatic lesions can cause complications, such as pain, spinal cord compression, bone fractures, and hypercalcemia [2]. Additionally, hospitalization for skeletal-related events (SREs) increases the financial burden of patients [3]. Osteoblasts and osteoclasts coordinate with one another to form stable bone remodeling. The invasion of cancer cells leads to the imbalance of homeostasis and ultimately to the destruction of bone, while changes in the bone microenvironment will further enhance the growth of cancer cells, forming a vicious cycle [4]. Osteoclast inhibitors mitigate SREs associated with bone metastasis [5], including bisphosphonates and denosumab, which are commonly used in clinical practice. Although these two osteoclast inhibitors exert some effect in clinical treatment, neither has affected survival outcomes [6]. Additionally, continuous and increased use may lead to long bone atypical fractures, osteonecrosis of the mandible, and infection risks [7, 8]. Therefore, it is of great importance to further develop effective drugs to reduce the incidence of SREs associated with bone metastasis in cancer.

An osteoclast is known to be a multinucleated and large cell, which is differentiated from macrophage/monocyte lineage cells by the receptor activator of nuclear factor-κB ligand (RANKL) and macrophage colony-stimulating factor (M-CSF) [9]. The binding of RANK to RANKL activates the nuclear factor of activated T cell cytoplasmic 1 (NFATc1), a key modulator of the formation of osteoclasts, further inducing the osteoclast-associated gene expression, including matrix metalloproteinase-9 (*MMP-9),* cathepsin K (*Ctsk*), and tartrate-resistant acid phosphatase (*TRAP*) [10, 11].

The NF-κB signaling pathway is a critical pathway in the differentiation of osteoclast. Many research reports have demonstrated that suppression of the NF-κB signaling pathway inhibits osteoclast formation and function [12–14]. The 3-phosphoinositide-dependent protein kinase 1 (*PDK1*) gene was first recognized as an essential upstream lipid kinase of the phosphatidylinositol 3-kinase/protein kinase B (PI3K/AKT) cascade in insulin signal transduction. Activation of AKT through phosphorylation triggers a cascade that further activates AKT downstream factors [15]. Most studies on *PDK1* have focused on its relation to tumors [16].

Osteoclasts, as the only bone resorptive cells in vivo, play important roles in osteolysis induced by tumors. Tumor cells in the bone microenvironment secrete several cytokines that trigger osteoclast activity, which in turn increases various lymph cytokines and growth factors that stimulate the tumor cell proliferation [17]. Interestingly, recent research reports have demonstrated that F2r responds to RANKL activation and impedes osteoclastogenesis by suppressing both the F2r-NF-κB and F2r-AKT signaling pathways [18]. Moreover, research has shown that stachydrine inhibiting osteoclastogenesis via AKT signaling prevents LPS-induced bone loss [19]. Since *PDK1* is an upstream activating element of AKT, we hypothesized that *PDK1* would affect bone resorption and the formation of osteoclasts through the AKT/NF-κB pathway.

## Materials and methods

### Materials and reagents

Fetal bovine serum (FBS), FITC‐conjugated phalloidin and DAPI stain, IRDye fluorescent-labeled secondary antibodies, and alpha modification of Eagle minimal essential medium (α‐MEM) were procured from Gibco (Thermo Fisher Scientific, Waltham, MA, USA). The TRAP staining kit and cell counting kit‐8 (CCK-8) were procured from Sigma‐Aldrich (St. Louis, MO, USA). ELISA kits against CTX‐1, TRACP‐5b, BGP, and PINP were procured from Cusabio Biotech (Wuhan, China). RANKL and M-CSF were bought from R&D Systems (Minneapolis, MN, USA). The specific primary antibodies, including PDK1 (rabbit, cat. no.: 5662S), p-AKT (phosphor T308; rabbit, cat. no.: 13038S), AKT (rabbit, cat. no.: 4685S), p-IκBα (rabbit, cat. no.: 2859S), IκBα (rabbit, cat. no.: 4812S), p-P65 (phosphor Ser536; rabbit, cat. no.: 3033S), P65 (rabbit, cat. no.: 8242S), RANK (rabbit, cat. no.: 4845S), and NFATC1 (rabbit, cat. no.: 5861S) were procured from Cell Signaling Technology (Danvers, MA, USA). The specific primary antibody, Ctsk (rabbit, cat. no.: ab187647), was purchased from Abcam (Cambridge, UK).

### Generation and identification of PDK1-cKO (RANK^Cre^. PDK1^flox/flox^) mice

**Fig. 1:**
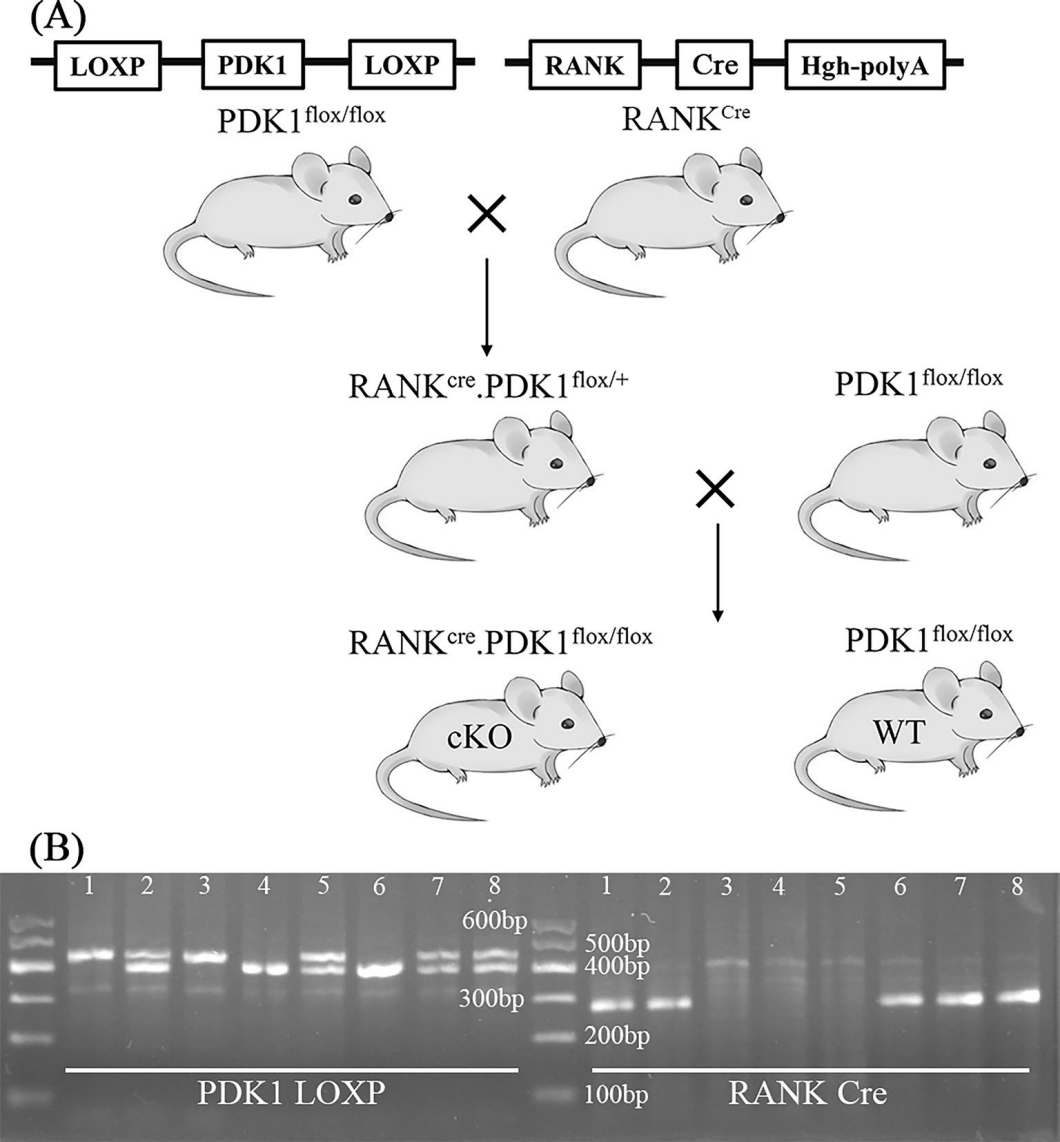
The acquisition and identification of osteoclast‐specific *PDK1* knockout in mice. **A** Flowchart of *PDK1* knockout in osteoclasts. **B** Identification results (1, RANK^Cre^. PDK1^flox/flox^; 2, RANK^Cre^. PDK1^flox/+^; 3, PDK1^flox/flox^; 4, PDK1^+/+^; PDK1, 3-phosphoinositide-dependent protein kinase-1)

PDK1^flox/flox^ and RANK^Cre^ mice were designed by GemPharmatech (Nanjing, China). Cre recombinase expression was regulated via RANK promoter transcriptional control. PDK1^flox/flox^ and RANK^Cre^ mice were mated to produce RANK^Cre^. PDK1^flox/+^ mice. RANK^Cre^. PDK1^flox/+^ and PDK1^flox/flox^ were mice mated to produce PDK1-cKO (RANK^Cre^. PDK1^flox/flox^) and wild-type (WT) (PDK1^flox/flox^) mice (Fig. 1A). Mice were kept in individually ventilated cages (temperature: 22–26 °C; humidity: 50–60%; dark/light: 12/12 h) with free to eat standard feed and freshwater. Mice were numbered with ear tags 2 weeks after birth. Mice tails were cut into 1 mm segments for PCR. DNA was acquired utilizing a TIANamp genomic DNA extraction kit (TIANGEN Biotech, Beijing, China) following the instructions stipulated by the manufacturer. Primers were provided by Shanghai Sangon Biological Engineering Technology (Shanghai, China) (Table 1). The PCR reaction system was as follows: 25 μL 2 × Tag MasterMix, 2 μL primer F, 2 μL primer F, 1 μL template, and 20 μL ddH_2_O. The PCR reaction conditions were set according to the 2 × Tag MasterMix instructions (Shanghai Sangon Biological Engineering Technology, Shanghai, China). The PCR products were used for horizontal agarose gel electrophoresis (Fig. 1B). Mice were weighed weekly and weights were recorded. All of the animal experimentations were undertaken in compliance with the American Veterinary Medical Association (AVMA) Guidelines for the Euthanasia of Animals (2020). Mice were anesthetized via of 2% sevoflurane in a special container, CO_2_ was then injected into the container at a rate that replaced 25% of container volume per minute. When it was confirmed that the mice were dead, the CO_2_ was turned off. The execution dates ranged from November 2019 to May 2021.

**Table 1:** Primers sequences for PCR

Genes	Primers sequences (5′ → 3′)	
*PDK1*	Forward	TGTGCTTGGTGGATATTGAT
	Reverse	AAGGAGGAGAGGAGGAATGT
*RANK Cre*	Forward	ACTTCTCCATGGTAGCCTCC
	Reverse	AATATGGGGGTGGGGTGATA
*Ctsk*	Forward	CTTCCAATACGTGCAGCAGA
	Reverse	TCTTCAGGGCTTTCTCGTTC
*TRAP*	Forward	CACTCCCACCCTGAGATTTGT
	Reverse	CCCCAGAGACATGATGAAGTCA
*MMP-9*	Forward	CCTGTGTGTTCCCGTTCATCT
	Reverse	ACCCGAATCTAGTAAGGTCGC
*NFATc1*	Forward	CCGTTGCTTCCAGAAAATAACA
	Reverse	TGTGGGATGTGAACTCGGAA
*β-Actin*	Forward	TCTGCTGGAAGGTGGACAGT
	Reverse	CCTCTATGCCAACACAGTGC

### Alizarin Red and Alcian Blue staining to visualize mice skeletons

At 8 weeks, WT and PDK1-cKO mice were euthanized, placed on a foam board in a prone position, and photographed utilizing an X-ray imager (Faxitron MX20/DC2; voltage: 5.0 kV; time of exposure: 6.0 s). Mice were observed for skeletal deformities and variation and then dissected after photography. The main viscera were dissociated to observe whether there was any variation. The whole skeleton was carefully dissociated and muscle tissue was removed as much as possible, and then the skeleton was fixed in 95% alcohol for 3 d, which was then digested in acetone for 48 h to remove excess adipose tissue. The skeleton was stained with pre-configured staining solution for 5 days (75% alcohol: 0.3% Alcian Blue: 0.1% Alizarin Red: glacial acetic acid = 1:1:1:17). After staining, the skeleton was transferred to 1% KOH and soaked for 48 h, and then placed in different solutions for 24 h (glycerol: 1% KOH = 1:4; glycerol: 1% KOH = 1:1; glycerol: 1% KOH = 4:1). After the muscle tissue was transparent, the staining results were observed.

### Murine model of prostate cancer-induced osteolysis

Anesthetization of 8‐week-old PDK1-cKO and WT male mice (*n* = 6) was done by inhalation of sevoflurane and preoperative IP injection of penicillin (200 U/g) to prevent infections. The needle was injected into the proximal tibia head of the right lower extremity with a micro-syringe, then rotated 2–3 mm. A 10 μL cell suspension containing 5 × 10^5^ RM-1 was slowly injected, then the syringe was removed, the site was disinfected with iodophor, and mice were placed back into their cages after waking up. After 2 weeks, the tibia of the right lower limb was fixed in 4% paraformaldehyde for micro-CT detection.

### Detection of bone conversion markers

8‐week-old mice venous blood was harvested and centrifuged for 20 min at 1600 rpm. Bone formation markers (PINP, BGP) and bone resorption markers (TRACP-5b, CTX-I) were detected using an ELISA kit (Wuhan Huamei Biological Engineering Co., Ltd., Wuhan, China) following the instructions stipulated by the manufacturer.

### Micro-CT scanning and 3D reconstruction of the tibia

2 weeks after the murine model of prostate cancer-induced osteolysis was established, the right tibia was taken for scanning and 3D reconstruction was conducted using a micro‐CT scanner (SkyScan1072) (voltage: 70 kV; current: 200 μA; resolution: 10 μm). The osteolysis area was randomly selected for quantitative analysis. The main analysis indexes were structure model index (SMI), connectivity density (Conn‐Dens, 1/mm^3^), trabecular separation (Tb. Sp, μm), trabecular thickness (Tb. Th, μm), trabecular number (Tb. N, 1/mm), and bone volume to tissue volume (BV/TV, %).

### TRAP and HE staining of the tibial bone

After micro-CT scanning, the tibial bones were decalcified in 10 percent ethylenediaminetetraacetic acid (EDTA) for a fortnight at a temperature of 4 °C ensured by continuous dehydration in 40 percent, 75 percent, and 95 percent ethanol for 60 min, followed by 2 dehydrations in 100 percent ethanol with each for 30 min. Tissue specimens were cleared in xylene for 15 min before being infiltrated with paraffin for 3 h. The sequential segments were split into 5 μm slices, followed by staining with HE and TRAP, and subsequently visualized utilizing an inverted light microscope (Nikon Eclipse TS100, Tokyo, Japan).

### Bone marrow-derived macrophage cell extraction and osteoclast differentiation

8-week-old PDK1-cKO and WT mice were euthanized. The intact femur and tibia were isolated after disinfection in 75% alcohol for 5 min. The ends of the femur and tibia were cut off, the α-MEM was drained with a 1-mL syringe, and the bone marrow cavity was rinsed 3 times. The collected rinse solution was sieved with a 200-μm sterile filter, then the supernatant was discarded and subjected to suspension in α-MEM comprising 10 percent FBS and M-CSF (25 ng/mL). The medium was transferred to a T-75 culture flask and cultured at a temperature of 37 °C with a CO_2_ concentration of 5%. The α-MEM comprising 10 percent FBS and M-CSF (25 ng/mL) was replaced at a frequency of once every 2 days. After 4 days, several bone marrow-derived macrophage cells (BMMs) were observed, which were digested and suspended in α-MEM with 10 percent FBS and M-CSF (25 ng/mL). BMMs were kept in 96-well plates (8000/well), then subsequently placed in a cell incubator for culturing. After 24 h, the α-MEM comprising 10 percent FBS, RANKL (100 ng/mL), and M-CSF (25 ng/mL) was used to replace the medium. Roughly 6 days after RANKL induction, multinucleated osteoclasts were observed under a microscope. After discarding the medium, rinse operation was done 2 times using PBS, and then 4% paraformaldehyde was added for fixation at room temperature (RT) for 30 min. The fixative was subsequently discarded and washing routine was conducted twice with PBS, and TRAP dye was added at RT for 1 h. Then, the TRAP dye was discarded, followed by 2 times rinse using PBS and air seasoning. Finally, pictures were taken and the number of osteoblasts was calculated.

### BMMs proliferation/viability assay

BMMs of PDK1-cKO and WT mice were plated in 96-well plates (6000/well) and cultured in α-MEM comprising M-CSF (25 ng/mL). Then, after 48 h, each well was added 10 μL CCK-8, followed by incubation at a temperature of 37 °C for 2 h, and values recording of OD at 450 nm utilizing a microplate reader. The proliferation activity of PDK1-cKO and WT mice was statistically analyzed according to the OD value.

### Podosome actin belt formation assay

BMMs were placed in 96-well plates (8 × 10^3^/well) to induce mature osteoclasts (same induction process as described above). When mature osteoclasts were observed, cells were gently rinsed twice using 1 × PBS, fixed using percent paraformaldehyde at RT for 10 min, and rinsed thrice using 1 × PBS; then cells were permeated using 0.1 percent Triton X-100 in PBS for 5 min at RT, blocked using percent BSA in PBS for 30 min, rinsed 2 times with 0.2 percent BSA, diluted with rhodamine-conjugated phalloidin in 0.2 percent BSA (1:100), incubated for 1 h at RT, rinsed 4 times using 0.2 percent BSA and 4 times using 1 × PBS, and stained using DAPI for 5 min. Lastly, photos were taken under a fluorescence microscope (Life Technologies, Carlsbad, CA, USA). The data were evaluated utilizing ImageJ (NIH, Bethesda, Maryland, USA).

### Hydroxyapatite resorption assay

BMMs were placed in 6-well plates (2 × 10^4^/well) and induced with α-MEM comprising 10 percent FBS, RANKL (100 ng/mL), and M-CSF (25 ng/mL). When the round-like preosteoclasts were observed under a microscope, they were digested with 0.25% trypsin and placed in a 96-well hydroxyapatite-coated bone absorption plate (2000/well). After 3 d of culturing, the hydroxyapatite coating was absorbed into a transparent area with irregular shape and the culture medium was sucked out. Wells were washed with 1 × PBS 3 times and washed with 5% sodium hypochlorite solution for 10 min to eliminate the remaining adherent cells. Finally, hydroxyapatite-coated bone absorption plates were washed using 1 × PBS thrice, visualized using an inverted microscope, and photographed after air drying. The absorption area was measured using ImageJ (NIH, Bethesda, Maryland, USA).

### Quantitative real-time PCR (qPCR)

The total RNA from PDK1-cKO and WT osteoclasts was obtained utilizing a TaKaRa MiniBEST universal RNA extraction kit (Takara Bio Inc, Kyodo, Japan) following the instructions stipulated by the manufacturer. The reverse transcription of RNA into cDNA was performed. The acquired cDNA was utilized as a template for qPCR, which was carried out on an ABI Prism 7500 system (Thermo Fisher Scientific, Waltham, MA). The PCR cycling setting was as illustrated below: 95 °C for 30 s; 40 cycles at 95 °C for 5 s; and 60 °C for 34 s. Table 1 lists the primers utilized in this research. The relative gene expression was evaluated utilizing the 2^−ΔΔCt^ method.

### Protein extraction and Western blot analysis

To investigate the impacts of conditional knockout of the *PDK1* gene on the early activation of RANKL signaling pathways, BMMs were treated with serum-free starvation for 3 h and subsequently triggered with RANKL (100 ng/mL) for 5, 10, 20, 30, or 60 min. To evaluate the effects of conditional knockout of *PDK1* on the late RANKL activated signal cascade, BMMs were triggered with RANKL (100 ng/mL) for 0, 2, 4, or 6 days; WT mice were used as the control. Total cellular proteins (TCPs) were obtained utilizing a TCP extraction kit (Sigma-Aldrich, St. Louis, MO, USA) as per the protocols stipulated by the manufacturer. TCPs were isolated by 10 percent SDS-PAGE gel and loaded onto nitrocellulose membranes (Thermo Fisher Scientific, Shanghai, China). Blocking of the membranes was done with 5 percent skim milk in 1 × TBST for 1 h at RT, followed by incubation using primary antibodies (for the dilution ratio, refer to the reagent instructions) for 15 h at 4 °C, and 3 times rinse with 1 × TBST. Afterward, membranes were subjected to incubation with IRDye fluorescent-labeled secondary antibodies at RT for 1 h. The corresponding protein bands were imaged using an LI-COR Odyssey SA Infrared Imaging Scanner. Densitometric analyses were measured using ImageJ (NIH, Bethesda, Maryland, USA).

## Statistical analyses

All of the data are presented as the mean ± standard deviation (SD). All the experimentations conducted in this study were replicated thrice unless otherwise noted. Statistical differences were determined using SPSS v22.0 (SPSS Inc., Chicago, IL, USA). To compare the two groups, an unpaired Student's t-test was employed. The significance threshold was *P* < 0.05.

## Results

### Effects on mice phenotypes after *PDK1* deletion in osteoclasts

**Fig. 2:**
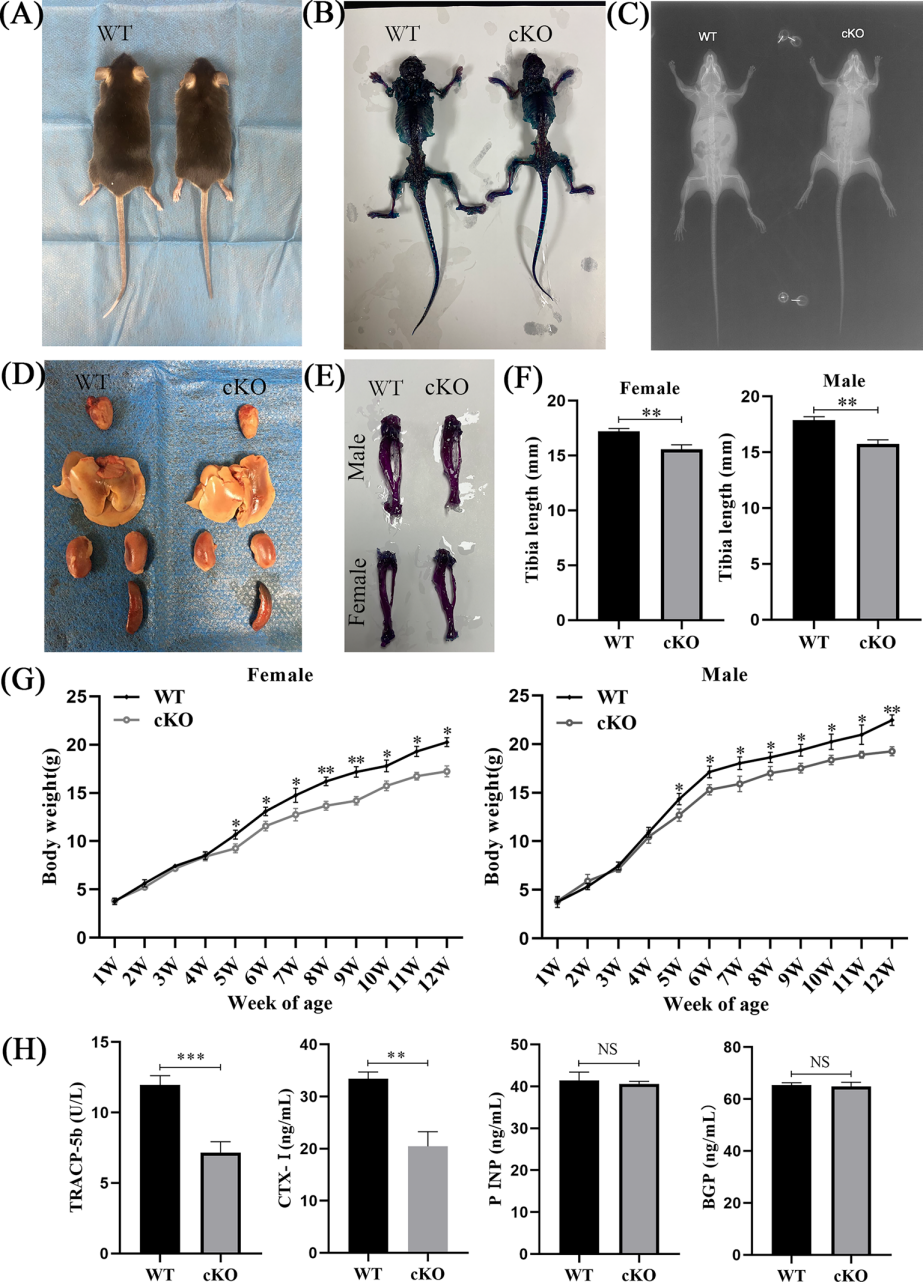
Effects on mice phenotypes after *PDK1* knockout in osteoclasts. **A** Gross anatomy. **B** staining of skeletal bone tissues using Alizarin Red and Alcian Blue. **C** X‐ray radiographs. **D** Vital organs of 8-week-old PDK1 cKO and WT mice. **E, F** Comparison of tibia length of PDK1 cKO and WT mice. **G** Body weight changes of 1- to 12-week-old PDK1 cKO and WT mice. **H** Comparison of bone resorption markers (TRAC5b, CTX-1) and bone formation markers (P1NP, BGP) in PDK1 cKO and WT mice. *N* = 6, all data are expressed as the mean ± SD. ^NS^*P* > 0.05, *P < 0.05, **P < 0.01, and ***P < 0.001. PDK1, 3-phosphoinositide-dependent protein kinase-1; WT, wild type; cKO, conditional knock out; TRACP‐5b, tartrate‐resistant acid phosphatase 5b; CTX‐1, C‐terminal telopeptide of type I collagen; PINP, procollagen I N‐terminal propeptide; BGP, bone Gla protein

Previous studies have shown that the whole-body *PDK1* knockout gene in mice contributes to early embryonic death [20]. Therefore, in this study, we established a mouse model for the conditional *PDK1* knockout in osteoclast cells. To evaluate the effects on the growth and development of mouse bone after *PDK1* deletion in osteoclasts, we monitored skeleton size and body weight. Results revealed that, when compared to WT mice, PDK1-cKO mice had smaller skeleton development and lower body weight after 5 weeks and onwards. The difference in body weight between the 2 groups was significant (*P* < 0.05) and no malformations were detected in the bone or vital organs of PDK1-cKO mice (Fig. 2A–G). To determine the functional level of osteoblasts and osteoclasts in vivo, we detected the contents of bone turnover markers in 8-week-old mice’s serum. Results revealed that bone resorption markers (TRACP-5b, CTX-1) of PDK1-cKO mice were significantly reduced when compared to WT mice (*P* < 0.01). However, there were no considerable variations between the contents of bone formation markers (P1NP, BGP) (Fig. 2H, *P* > 0.05).

### Osteoclast‐specific knockout of *PDK1* ameliorated prostate cancer-induced osteolysis in vivo

**Fig. 3:**
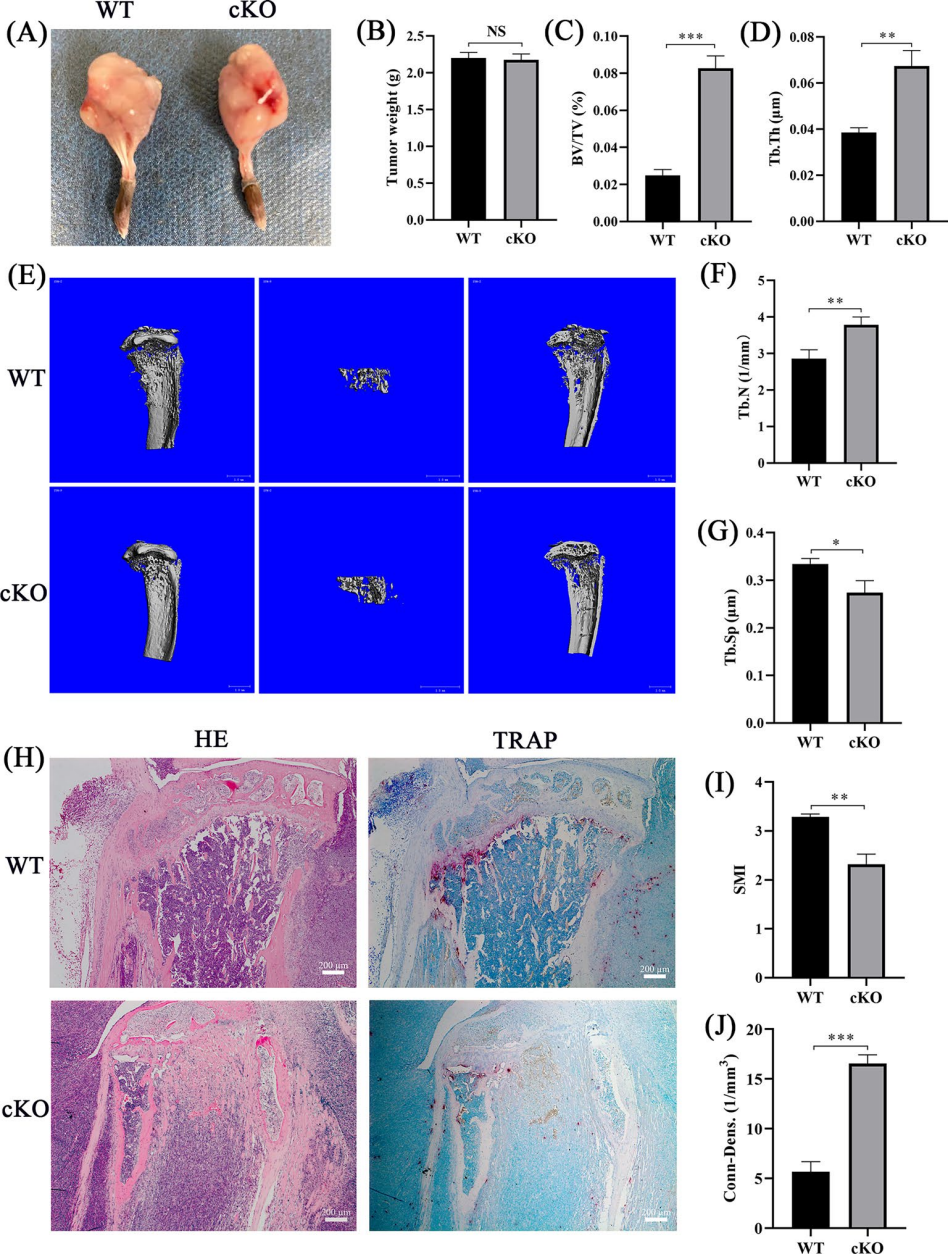
Effects of osteoclast‐specific knockout of *PDK1* on prostate cancer-induced osteolysis. **A** Anatomy of bone metastases from prostate cancer in PDK1 cKO and WT mice. **B** Comparison of tumor mass between PDK1 cKO and WT mice. **C, D, F, G, I, J** Bone parameter analysis. SMI, structural model index; Conn-dens., connectivity density; Tb. Sp, trabecular separation; Tb. N, trabecular number; Tb. Th, trabecular thickness; BV/TV, bone volume fraction. **E** Three-dimensional reconstruction of the tibia; scale bar = 1 mm.** H** HE and TRAP staining of tibia sections; scale bar = 200 μm. *N* = 6, all of the data are presented as the mean ± SD. ^NS^*P* > 0.05, *P < 0.05, **P < 0.01, ***P < 0.001

To evaluate the impacts of osteoclast‐specific knockout of *PDK1* on prostate cancer-induced osteolysis, we utilized a mouse model of prostate cancer-induced osteolysis. Results revealed that there was no considerable variation in tumor mass between WT and PDK1-cKO mice (Fig. 3A, B *P* > 0.05). However, osteolysis in PDK1-cKO mice was reduced when compared WT mice (Fig. 3E). Additional analysis of bone parameters showed that when compared to WT mice, BV/TV, Tb. Th, Tb. N, and Conn-dens. significantly increased (Fig. 3C, D, F, G *P* < 0.05), whereas Tb. Sp and SMI reduced (Fig. 3I, J *P* > 0.05). In the bone tissue sections, the number of trabeculae in PDK1-cKO mice was higher than in WT mice after HE staining; this result is consistent with the bone parameter analysis. Moreover, the number of TRAP-positive osteoclasts in PDK1-cKO mice was lesser as opposed to that in WT mice after TRAP staining (Fig. 3H).

### Osteoclast‐specific knockout of *PDK1* suppressed RANKL-induced osteoclastogenesis, podosome belt formation, and bone resorption function in vitro

**Fig. 4:**
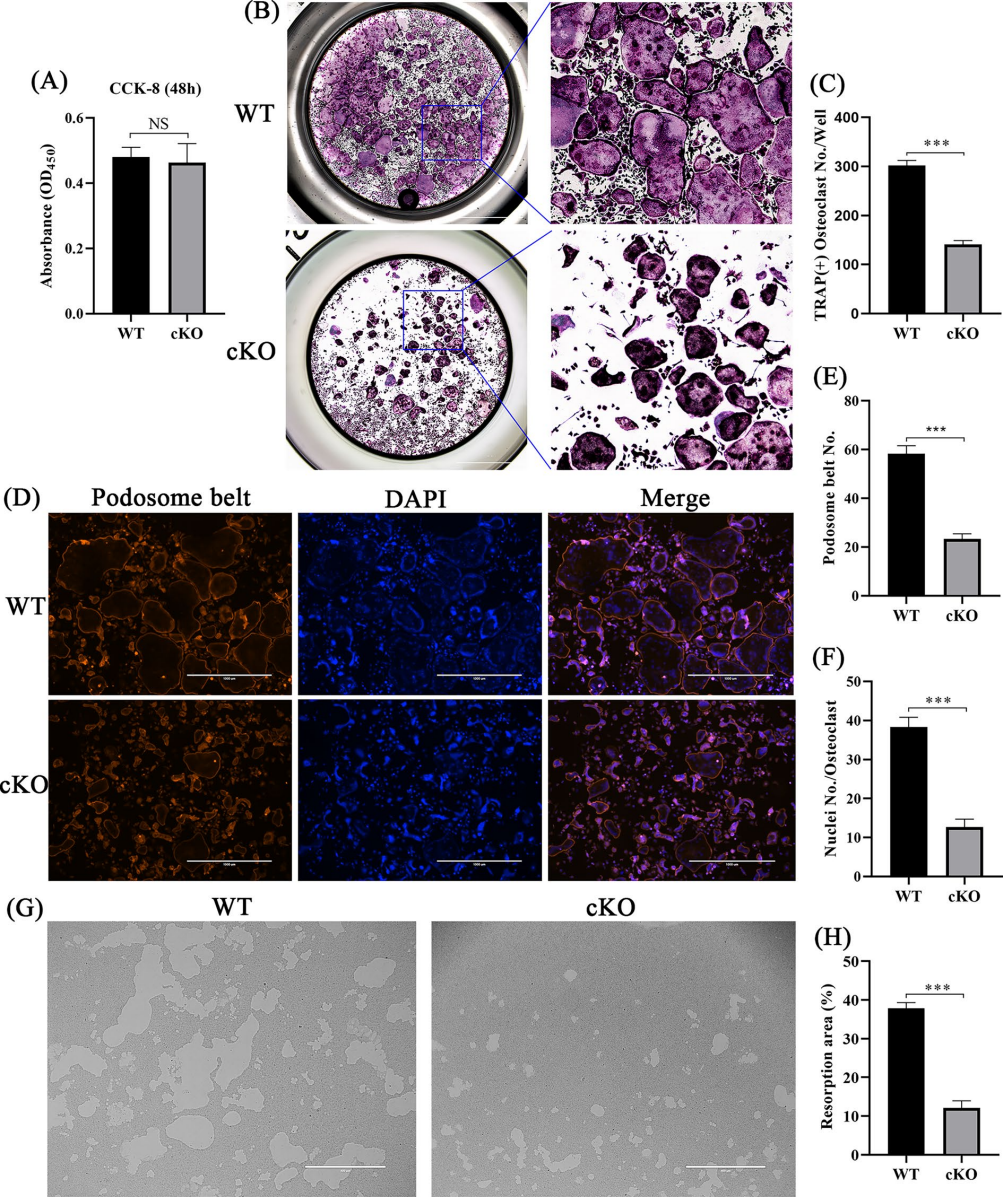
Osteoclast‐specific knockout of *PDK1* suppressed RANKL-stimulated osteoclastogenesis, bone resorption function, and podosome belt formation in vitro. **A** BMM viability detected by CCK-8 after 48 h of M-CSF stimulation. **B** Images of mature osteoclasts after TRAP staining; scale bar = 2000 μm. **C** Quantification of osteoclasts in PDK1 cKO and WT mice. **D** Fluorescence images of the effects of *PDK1* on the podosome actin belt; scale bar = 1000 μm. **E** Count of osteoclasts with podosome actin belts. **F** Quantification of the average number of nuclei per osteoclast. **G** Images showing hydroxyapatite resorption; osteoclasts were seeded on hydroxyapatite-coated bone absorption plates and stimulated with RANKL. **H** Quantification of resorbed hydroxyapatite. *N* = 3, all data are expressed as the mean ± SD. *P < 0.05, **P < 0.01, and ***P < 0.001. BMMs, bone marrow-derived macrophage cells; TRAP, tartrate-resistant acid phosphatase

To explore the impacts of osteoclast‐specific knockout of *PDK1* on the proliferation activity of BMMs, after 48 h of M-CSF stimulation, we examined the proliferative activity of BMMs. Results revealed that there was no considerable variation between WT and PDK1-cKO mice (Fig. 4A, *P* > 0.05). To further explore the effects of osteoclast‐specific knockout of *PDK1* on RANKL-induced osteoclastogenesis, on the sixth day of RANKL induction, TRAP staining was applied to mature osteoclasts. Results revealed that conditional knockout of *PDK1* in osteoclasts inhibited the differentiation of RANKL‐induced osteoclasts (Fig. 4B). The count of TRAP‐positive multinucleated cells (*n* ≥ 3) in PDK1-cKO mice was lesser as opposed to that in WT mice (Fig. 4C, *P* < 0.001). To examine morphological alterations and podosome belt formation, mature osteoclasts were subjected to staining with rhodamine-phalloidin. When compared to WT mice, there were smaller osteoclasts with fewer nuclei in PDK1-cKO mice (*P* < 0.001), suggesting that the knockout of *PDK1* in osteoclasts inhibited precursor cell fusion (Fig. 4D–F).

Given that osteoclast‐specific knockout of *PDK1* impaired the formation of podosome actin belt, which is a precondition for osteoclast function, we postulated that osteoclast‐specific knockout of *PDK1* would also inhibit osteoclast bone resorption. We used hydroxyapatite-coated bone absorption plates to explore whether the deletion of *PDK1* in osteoclasts would have an effect on osteoclast resorption function. Results revealed a smaller resorption area after *PDK1* deletion in osteoclasts (Fig. 4G, H, *P* < 0.001). These findings indicated that the *PDK1* deletion in osteoclasts can effectively inhibit osteoclast bone resorption function.

### Conditional knockout of *PDK1* in osteoclasts inhibited osteoclast-specific gene expression

**Fig. 5:**
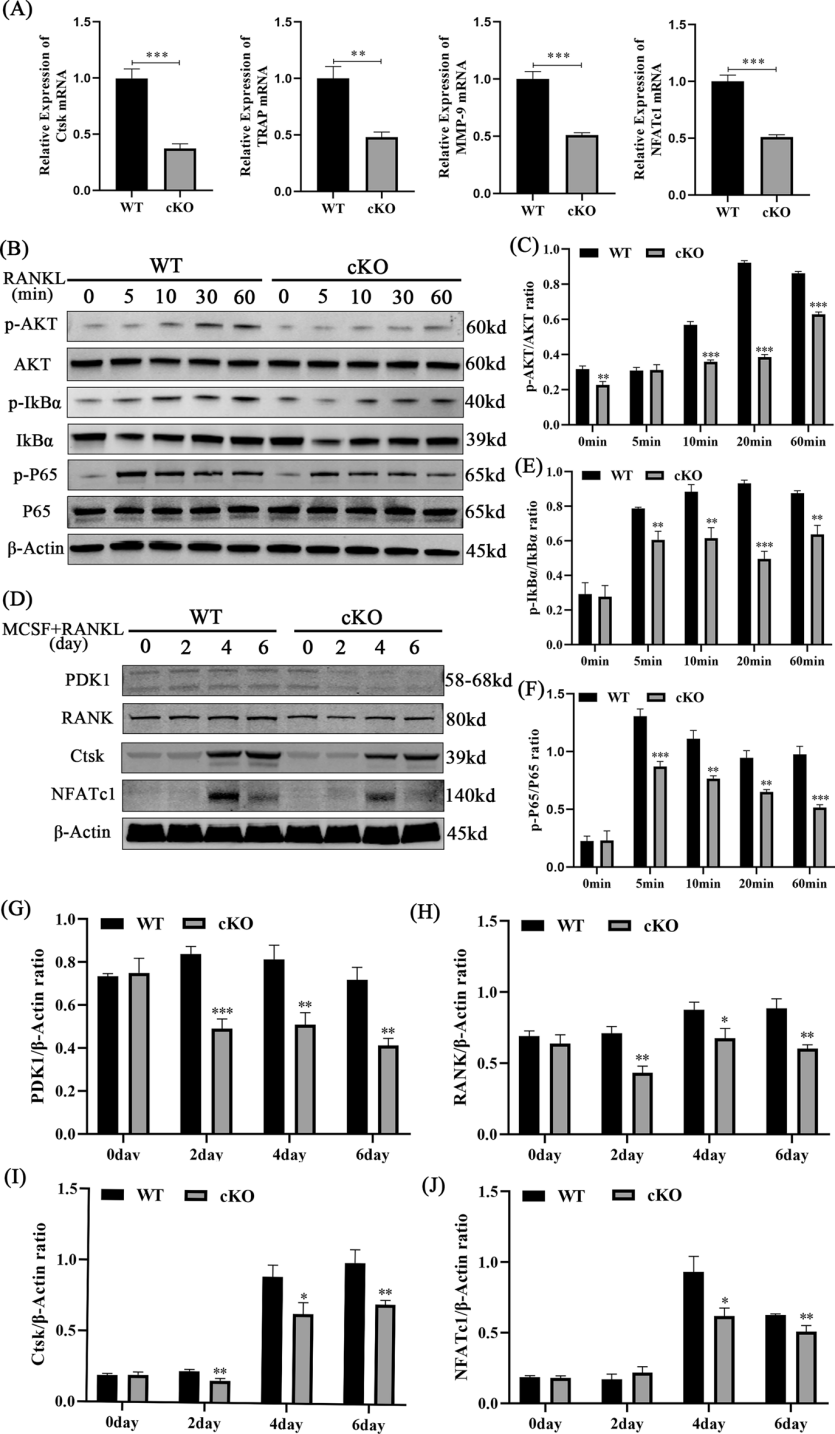
Deletion of *PDK1* in osteoclasts inhibited RANKL‐stimulated expression of osteoclast-specific gene and NF‐κB activities. **A** Relative mRNA expression levels of *Ctsk*, *TRAP*, *MMP-9*, and *NFATc1*; mRNA expression was normalized to WT mice. **B** BMMs were stimulated with RANKL for 0, 5, 10, 30, or 60 min; total cell proteins (TCPs) were obtained and the expression of p-AKT, AKT, P65, p-P65, IκBα, and p-IκBα was detected by Western blot. **C, E, F** Relative ratios of phosphorylated/unphosphorylated proteins. **D** BMMs were triggered with RANKL for 0, 2, 4, or 6 days; TCPs were extracted and the expressions of p-AKT, AKT, P65, p-P65, IκBα, and p-IκBα were identified by Western blot. **G, H, I, J** Relative expression of *PDK1*, *Ctsk*, *RANK*, and *NFATc1*. *N* = 3, all data are expressed as the mean ± SD. *P < 0.05, **P < 0.01, ***P < 0.001. BMMs, bone marrow-derived macrophage cells; PDK1, 3-phosphoinositide-dependent protein kinase-1; Ctsk, cathepsin K; NFATc1, nuclear factor of activated T cells; RANK, receptor activator for nuclear factor-κB

Upon the stimulation of osteoclast differentiation during BMM differentiation in osteoclasts, several osteoclast-specific genes (Ctsk, TRAP, MMP-9, NFATc1) are upregulated in BMMs [21, 22]. To further explore the underlying mechanisms, the osteoclast-specific genes expression was examined at the mRNA level. Results revealed that, when compared to WT mice, the relative expression levels of *Ctsk*, *TRAP*, *MMP-9*, and *NFATc1* were considerably inhibited in PDK1-cKO mice (Fig. 5A, *P* < 0.01).

### Deletion of *PDK1* in osteoclasts suppressed the RANKL-induced NF-κB signaling pathway

The NF-κB pathway is the main signaling pathway triggered during osteoclast formation [23]. To investigate the mechanisms that underlie the suppression impacts of the osteoclast‐specific knockout of *PDK1* on early osteoclastogenesis, the NF-κB signaling pathways in osteoclasts were identified by Western blot analysis. Seeding of the BMMs was done on 6-well plates and cultured over the night to enable cells to attach to the wall. Then, the cells were activated by RANKL for 0, 5, 10, 30, or 60 min, TCPs were extracted, and the expressions of p-AKT, AKT, P65, p-P65, IκBα, and p-IκBα were examined. Results revealed that when compared to WT mice, p-AKT/AKT, p-I κBα/IκBα, and p-P65/P65 declined in PDK1-cKO mice (Fig. 5B, C, E, F). To better examine the mechanisms that underlie the suppression effects of osteoclast‐specific knockout of *PDK1* on late osteoclastogenesis, TCPs were extracted after BMMs were induced by RANKL for 0, 2, 4, or 6 days. Results revealed that, when compared to WT mice, PDK1, Ctsk, RANK, and NFATc1 protein expression was considerably inhibited in PDK1-cKO mice (Fig. 5D, G–J).

## Discussion

Bone metastasis is clinically difficult to treat due to pain, increased SREs, decreased quality of life, and decreased overall survival, and there is currently no effective treatment [24]. Approved therapeutic agents for treating bone metastasis in the 2019 National Comprehensive Cancer Network (NCCN) Clinical Practice Guidelines in Oncology focus on the treatment of obvious pain and SREs. These drugs mainly include bisphosphonates, which inhibit the bone resorptive function of osteoclasts by triggering apoptosis, and denosumab, which is a RANKL-specific inhibitor that inhibits osteoclast activation and development, reduces bone resorption and increases bone mineral density [25]. Although bisphosphonates are now widely used in clinical practice, there is a risk of osteonecrosis and potential esophageal tumors after long-term use [26]. Therefore, identifying novel bone‑protective mechanisms is a pertinent area of research. Activated osteoclasts play key roles in tumor-related bone destruction through bone resorption [27]. RANKL produced by tumor cells stimulates osteoclast precursor cells to differentiate osteoclasts [28, 29], which thereby activates osteoclasts, further providing a suitable bone microenvironment for tumor growth [30, 31]. R Chen et al. found that N-CDs effectively abrogated RANKL-induced osteoclastogenesis. And, the in vivo administration of N-CDs in mice protected them against breast cancer cell-induced tibial bone loss [32]. Thus, the inhibition of osteoclasts is an important research direction for reducing SREs.

The present study demonstrated that the osteoclast‐specific knockout of *PDK1* ameliorated prostate cancer-induced osteolysis and reduced bone resorption markers in the blood in vivo. There was no considerable alteration in tumor mass; however, the activation of osteoclasts stopped the dormancy of tumor cells and promoted their growth [30, 33]. A possible explanation for this result is that the inhibition of osteoclasts was mainly manifested due to certain conditions within the bone microenvironment. The proliferation of tumor cells resulted in tumor tissue covering the tibia, then the diameter of the tumor far exceeded the diameter of the tibia, and osteoclasts were confined to bone tissue; thus, the inhibition of osteoclasts did not change tumor weight. In this research, we found that the conditional knockout of *PDK1* in osteoclasts in vivo led to smaller body size of mice. We used a RANK promoter‐driven Cre‐LoxP system to conditionally delete the *PDK1* gene in osteoclasts. When cells expressed RANK, Cre recombinase knocked out *PDK1*. However, RANK is not specific to the expression of osteoclasts and other cells are expressed in a small amount [34, 35]. Therefore, the small size of mice is likely due to the knockout of *PDK1* in other cells. Another surprising finding was that that the TRAP-positive osteoclast-like cells in tumor tissue were observed after histological TRAP-staining. This result is likely due to the scattered tumor-associated macrophages that were differentiated into osteoclasts under the activation of RANKL secreted by tumor cells, which is consistent with the observation of osteoclast-like cells in the soft tissue of leiomyosarcoma by Gibbons et al. [36].

**Fig. 6:**
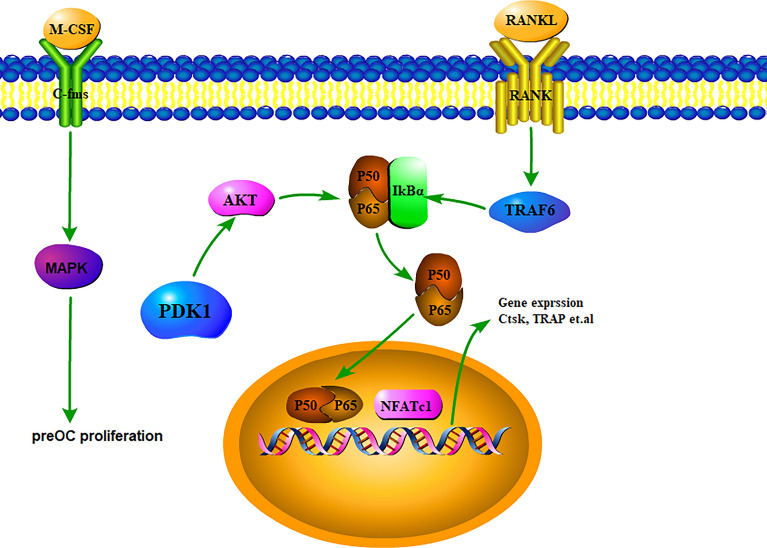
The mechanisms of *PDK1* in osteoclast formation. *PDK1* positively regulated osteoclast formation through the RANKL-induced NF-κB signaling pathway. M-CSF, macrophage colony stimulating factor; *PDK1*, 3-phosphoinositide-dependent protein kinase-1; MAPK, mitogen‐activated protein kinase; NF‐κB, nuclear factor κ‐light‐chain enhancer of activated B cells; NFATc1, nuclear factor of activated T cells 1

In this study, it was verified in vitro that the osteoclast‐specific knockout of *PDK1* suppressed RANKL-induced osteoclastogenesis, osteoclast-specific gene expression, and bone resorption function but the proliferation of BMMs was not affected. This result may be due to the RANK promoter‐driven Cre‐LoxP system that conditionally deleted the *PDK1* gene in osteoclasts. When cells expressed RANK, Cre recombinase knocked out *PDK1*; without M-CSF and RANKL stimulation, BMMs rarely expressed RANK. At this time, *PDK1* was not knocked out or was rarely knocked out in osteoclasts. Further investigation of the molecular mechanisms demonstrated that the deletion of *PDK1* in osteoclasts inhibited osteoclastogenesis via the RANKL-induced NF-κB signaling pathway (Fig. 6).

Osteoclasts are the only cells derived from macrophage/monocyte lineage cells with bone absorption functions in the body [37]. The proliferation, differentiation, and activation of osteoclasts require the participation of M-CSF and RANKL. MCS-F stimulates BMMs that become osteoclast precursors, and the RANKL binding to its receptor, RANK, stimulates the differentiation of osteoclast precursors into osteoclasts [38]. The NF-κB signaling pathway is an essential pathway for the differentiation of osteoclast. RANK signals recruit tumor necrosis factor receptor-associated factor 6 (TRAF6), which activates the mitogen-activated protein kinases (MAPKs), NF-κB and activator protein-1 (AP-1), which activate NF-κB that induces NFATc1, a key osteoclastogenesis regulator [39, 40]. Previous studies found that ZBTB20 positively regulated NF-κB activation and M1 polarization as well as the production of TGN-derived tubular carriers in BMDMs, playing a positive role in macrophage activation and mouse cranial osteolysis induced by Tips [41]. *PDK1* expression is dysregulated in many cancer types and is an interesting and unexplored target for cancer therapy [15, 42]. The PDK1 protein activates the PI3K–AKT pathway. Previous studies demonstrated that AKT actives the NF-κB signaling pathway in tumor cells [43, 44]. Q Jiang et al. demonstrated that prostate cancer patients associated mutations of SPOP impaired PDK1 degradation and thus activated the AKT kinase, resulting in tumor malignancies. PDK1 phosphorylation, could markedly evade SPOP-mediated PDK1 degradation, and played potently oncogenic roles via activating the AKT kinase [45]. AKT has also been found to enhance osteoclast formation and osteolysis in osteolysis-related diseases, PDK1 plays an important role as an upstream element of AKT. It is encouraging that similar results were observed in the early knockdown of PDK1 in osteoclast differentiation [46, 47]. Because RANK is expressed earlier than Ctsk during osteoclast differentiation, this experiment verified the effect of PDK1 knockout at an early stage of osteoclast differentiation. Previous studies found that complement C3a activates RANKL-induced osteoclastogenesis and bone resorption by regulating the PI3K/PDK1/SGK3 pathway [48]. Our study demonstrated that *PDK1* can further activate the NF-κB signaling pathway in osteoclasts by activating AKT. These results suggested that targeting the *PDK1* gene in osteoclasts might be a good treatment approach for prostate cancer-related osteolysis.

In our animal model, instead of injecting tumor cells into the circulatory system, we injected tumor cells specifically into the tibia's bone marrow cavity. Although this did not mimic distant metastasis in tumor cells, it directly led to bone damage. We did not further verify the above experimental results through *PDK1*-specific inhibitors, as there is no *PDK1*-specific inhibitor for only osteoclasts on the market. Moreover, we were concerned that *PDK1*-specific inhibitors would affect tumor cells at the time of intervention and that we could not properly evaluate the function of *PDK1* in osteoclasts.

To summarize, this research illustrated that the conditional knockout of *PDK1* in osteoclasts ameliorated prostate cancer-induced osteolysis effectively by suppressing RANKL-induced bone resorption and osteoclastogenesis.

## Conclusions

In general, our research indicated that conditional knockout of *PDK1* in osteoclasts in vitro ameliorated prostate cancer-stimulated osteolysis. *PDK1* deletion in osteoclasts in vivo suppressed osteoclast differentiation and bone resorption via the *PDK1*/AKT/NF-κB signaling pathway. These findings offer a new perspective for basic and clinical study of prostate cancer-induced osteolysis.

## Data Availability

The corresponding author can provide datasets created in this work upon reasonable request.

## Abbreviations

*PDK1*: 3-Phosphoinositide-dependent protein kinase-1
WT: Wild type
cKO: Conditional knock out
BMMs: Bone marrow-derived macrophage cells
α-MEM: Alpha modification of Eagle’s medium
BV/TV: Bone volume/tissue volume
Tb. Th: Trabecular thickness
M-CSF: Macrophage colony stimulating factor
SMI: Structural model index
Tb. Sp: Trabecular separation
Tb. N: Trabecular number
Conn-dens.: Connectivity density
Ctsk: Cathepsin K
NFATc1: Nuclear factor of activated T cells
micro-CT: Microcomputed tomography
HE: Hematoxylin and eosin
TBST: Tris-buffered saline–Tween 30
RANK: Receptor activator for nuclear factor-κB
TRAP: Tartrate-resistant acid phosphatase
NF-κB: Nuclear factor κB
RANKL: Receptor activator for nuclear factor-κB ligand
